# Implications of serological, genomic, and epidemiological insights into *Mycobacterium tuberculosis* mixed infection in a human-managed African elephant (*Loxodonta africana*)

**DOI:** 10.3389/ftubr.2024.1484394

**Published:** 2025-01-20

**Authors:** Giovanni Ghielmetti, Tanya J. Kerr, Johannes Loubser, Jennie Hewlett, Anzaan Dippenaar, Andre G. Loxton, Robin M. Warren, Wynand J. Goosen, Michele A. Miller

**Affiliations:** ^1^Division of Molecular Biology and Human Genetics, Faculty of Medicine and Health Sciences, South African Medical Research Council Centre for Tuberculosis Research, Stellenbosch University, Cape Town, South Africa; ^2^Section of Veterinary Bacteriology, Vetsuisse Faculty, Institute for Food Safety and Hygiene, University of Zurich, Zurich, Switzerland; ^3^National Zoological Gardens, Pretoria, South Africa; ^4^Department of Family Medicine and Population Health, Faculty of Medicine and Health Sciences, University of Antwerp, Antwerp, Belgium

**Keywords:** African elephant, mixed infection, *Mycobacterium tuberculosis*, tuberculosis, whole genome sequencing

## Abstract

Zoonotic and reverse zoonotic tuberculosis pose a risk to human and animal health, especially when individuals are in close contact. Whole genome sequencing (WGS) has led to significant advancements in our comprehension of bacterial disease dynamics, particularly regarding the transmission of pathogens at the population and individual levels. *Mycobacterium tuberculosis* was cultured from respiratory samples, including ante-mortem trunk wash, bronchoalveolar lavage, and post-mortem lung tissue samples of one African elephant (*Loxodonta africana*) euthanized in a South African zoo. The elephant presented with chronic weight loss and lethargy. Animal-side serological testing (Chembio DPP^®^ VetTB for Elephants) conducted on elephant serum yielded a positive result before euthanasia. At post-mortem examination, signs of chronic pneumonia and extensive macroscopic lesions compatible with tuberculosis were observed, confirming the presence of the disease. Genomic DNA was extracted from liquid MGIT culture and an improved culture medium (TiKa) and subjected to WGS analysis. Using a bioinformatic approach, the study identified a mixed infection involving two distinct strains of *M. tuberculosis*. The predominant strain was classified as lineage 1 and a second strain was identified as lineage 4. Both lineages have been found in a significant proportion of human tuberculosis cases in South Africa. No mutations associated with drug resistance were detected. The report highlights the susceptibility of elephants to human pathogens, particularly in high-burden settings. Biosafety challenges associated with handling and diagnosing tuberculosis in human-managed elephants are reported. We emphasize the importance of implementing effective preventive measures to ensure the safety of both humans and animals in zoo environments. Finally, the importance of multiple sampling and analysis of within-host mycobacterial populations for investigations of transmission is demonstrated.

## Introduction

Zoonotic and reverse zoonotic tuberculosis (TB) pose a risk to human and animal health, especially when individuals are in close contact (1). *Mycobacterium tuberculosis* is a clonal pathogen primarily known for causing TB in humans, where it has been shown to co-evolve for millennia (2). Historically, *M. tuberculosis* has been considered as not being maintained in domestic and wild animal populations, and its isolation from other hosts has been retained as anecdotal spillover events. Recent evidence, however, challenged this notion, suggesting potential consequences for animal conservation if *M. tuberculosis* were to become established in endangered animal species, particularly in human-managed animals living in close contact and potentially under stressful conditions (3, 4). The occurrence of reverse zoonotic transmission events, where *M. tuberculosis* is transmitted from humans to animals, has been suspected in various human-managed settings, particularly among Asian (*Elephas maximus*) and African elephants (*Loxodonta africana*) and cattle (5–8). Finally, transmission events of *M. tuberculosis* in free-ranging elephant populations have been reported, including a recent case observed in Kruger National Park (9).

Whole-genome sequencing (WGS) has led to significant advancements in our comprehension of bacterial disease dynamics, particularly regarding the transmission of pathogens at the population and individual levels. The phylogeny of *M. tuberculosis* currently consists of nine recognized lineages (L1–9), which consist of different strain types that may vary in their propensity to transmit and cause severe disease (2, 10, 11). Within the four major lineages (L1–4), two lineages (L2, L4) have been intensively characterized in taxonomic and phylogeographic studies (12, 13), while two others (L1 and L3) were the subject of more recent investigations (14–16). With the emergence of next-generation sequencing (NGS), the resolution of molecular tools has reached its zenith. This advancement enables the distinction between mixed-species infections and mixed-strain infections. In the former, different species belonging to the same genus simultaneously infect a single host, while in the latter, clearly defined strains belonging to the same species are found concurrently within the same host (17). The adaptation and phylogenetic evolution of *M. tuberculosis* strains are believed to be driven, among other extrinsic factors, by the host's immune system, contributing to the development of heterogeneous bacterial populations sharing the same biological niche. In humans, there is growing recognition that within-host mixed strain infections (MSIs) play a significant role in the development of tuberculosis (TB) drug resistance. Heteroresistance, characterized by the coexistence of both susceptible and resistant strains, is a key factor in this process (18). Without precise strain and drug resistance profiling, tracing TB transmission for epidemiological purposes and providing effective treatment for MSI patients becomes challenging. One significant knowledge gap lies in understanding the mechanisms of within-host evolution and interactions between different *M. tuberculosis* strains during mixed infections (19). Such interactions could influence disease severity, transmission dynamics, and the effectiveness of control measures (18). Recently, various bioinformatic tools have been developed to investigate TB MSIs, reflecting the scientific community's growing need to understand the mechanisms underlying this complex infection status (18, 20–22). As a result, whole genome sequences that once had to be discarded due to low quality can now be analyzed using advanced bioinformatic pipelines designed to handle this type of data.

The objective of this study was to investigate and report the serological, genomic, and epidemiological characteristics of a human-managed African elephant affected by tuberculosis. We address the diagnostic challenges, particularly regarding the analysis of *M. tuberculosis* whole genome sequences that exhibit a high number of heterozygous sites, utilizing a combination of recently developed state-of-the-art bioinformatics tools and models designed for human TB epidemiological and surveillance studies. The data originated from a zoo-kept African elephant in a country with a high burden of tuberculosis.

## Materials and methods

### Case description and monitoring

A female elephant was captured from the wild in Zimbabwe (origin unknown) in 1984 when she was estimated to be 3 years old. She was subsequently part of two different private circuses in South Africa until 2001, after which she was relocated to the National Zoological Gardens, Pretoria, South Africa. Due to potential exposure to human pathogens, including *M. tuberculosis*, blood samples were repeatedly collected in 2015, 2016, and 2018 for a series of monitoring assays, including serum for Dual Path Platform (DPP^®^) VetTB assay (Chembio Diagnostic Systems, Medford, NY, USA), which detects antibodies to specific *M. tuberculosis* complex antigens [ESAT-6, CFP10, and MPB83; (23)]. In 2018, at the age of 37, the elephant was euthanized based on deteriorating general condition and the DPP^®^ VetTB result, which was compatible with TB.

### Sample collection and processing

The elephant was immobilized using 12 mg etorphine and 60 mg azaperone (total doses) on the day of euthanasia for a clinical examination. A flexible endoscope was passed through the endotracheal tube, revealing a significant amount of thick cream-colored exudate in the trachea and bronchi, along with blood-tinged froth. Due to the material's viscosity, aspiration through the scope was challenging. A total of 150 ml sterile saline was instilled to aid in flushing out the material, and samples were collected by flushing the endotracheal tube with sterile saline. These samples were processed through centrifugation (2,000 × g for 30 min) to concentrate cellular material, and smears were acid-fast stained to detect the presence of mycobacteria. Due to the extent of the lesions observed via bronchoscopy and the zoonotic risk posed by the elephant to the zoo staff, the immobilized animal was euthanized with a headshot by an experienced marksman according to the AVMA guidelines for the euthanasia of animals (24). Additionally, lung tissue samples presenting macroscopic lesions compatible with tuberculosis were collected at post-mortem.

### Molecular analysis

The collected samples [bronchioalveolar lavage (BAL), endotracheal tube (ETT), trunk wash (TW) fluid, and lung tissue] were screened for the presence of *M. tuberculosis* complex DNA using the GeneXpert MTB/RIF Ultra assay (Cepheid, Sunnyvale, CA, USA), as previously described (25, 26). Briefly, lung tissue homogenate and respiratory samples were directly mixed at a ratio of 2:1 with the Xpert lysis buffer containing NaOH and isopropanol (Cepheid) and incubated for 15 min with constant shaking under BSL3 conditions. Thereafter the mixtures were loaded into the sample chamber of the cartridge for automatic processing and analysis.

### Culture and speciation

In parallel, the collected samples were processed and inoculated into conventional liquid culture media (BACTEC™ MGIT, Becton Dickinson, Franklin Lakes, NJ, USA) supplemented with growth supplement (oleic acid, albumin, dextrose, catalase; OADC), as well as polymyxin B, amphotericin B, nalidixic acid, trimethoprim, and azlocillin (PANTA; Becton Dickinson) and the improved mycobacterial culture method (TiKa Diagnostics, London, UK), as previously reported (27). Following an indication of growth positivity, each liquid culture was inspected for acid-fast bacilli after Ziehl-Neelsen (ZN) staining. All culture-positive crude extracts were subjected to speciation using a PCR targeting genetic regions of difference (RD) as previously described (28). Thereafter, 1 mL of two positive liquid cultures derived from ETT, one conventional MGIT and one TiKa liquid culture, were further inoculated into 5 mL Middlebrook 7H9 (Thermo Fisher Scientific, Waltham, MA, USA) media and incubated for an additional 21 days at 37°C for DNA extraction.

### DNA extraction and whole genome sequencing

Genomic DNA was extracted from heat-inactivated (98°C for 45 min) culture pellets and WGS was performed as previously described (29). Briefly, libraries were prepared with the Nextera DNA Flex Library Prep Kit (Illumina, San Diego, CA, USA). The quality of each library was assessed with a FragmentAnalyzer Automated CE System (Agilent, Santa Clara, CA, USA) using a Next Generation Sequencing Fragment Kit (1–6,000 bp; Agilent). Libraries were paired-end sequenced in 250 bp reads on an Illumina MiSeq System using the MiSeq Reagent Kit [version 3, 600-cycle; (30)]. All generated data were deposited at the European Nucleotide Archive (ENA; BioProject PRJEB79262).

### Whole genome sequence analysis

The FastQ files containing the raw paired-end reads were processed using automated pipelines for *M. tuberculosis* NGS analysis (31, 32). Briefly, MTBseq v_1.0.4 was employed with standard parameters to infer general metrics, including the total number of reads, percentage of mapped reads, GC-content, and mean coverage using *M. tuberculosis* H37Rv as reference. Thereafter, TBProfiler v_ 6.3.0 [profile] was employed to predict lineage and drug resistance using the curated tbdb database (https://github.com/jodyphelan/tbdb). Finally, to estimate the frequency of the lineage- and sub-lineage-specific alleles for all known positions listed in tbdb, the TB-profiler [lineage] script was employed on generated binary alignment map (BAM) files using the –snps option. Genomic positions showing ≥ 1 read with lineage-specific allele were filtered to estimate the fraction of each strain present in the whole genome sequences. Using the BAM files and Artemis (v18.1.0), 10 randomly chosen genomic positions were inspected manually to confirm the accuracy of the (sub-) lineage markers identified. In conjunction with the TBProfiler pipeline, a statistical tool designed to compare sequences by applying a Gaussian Mixture model (GMM) approach to distinguish different strain lineages' fractions and drug resistance profiles in *M. tuberculosis* MSIs, was used to confirm the estimated mixing proportions (18).

## Findings

Seroconversion occurred between 2016 and 2018, as the serology DPP^®^ VetTB assay yielded negative results in 2015–2016 but a positive outcome for the presence of circulating CFP10/ESAT-6-reactive antibodies in 2018, while no MPB83 antibodies were detected (Table 1).

**Table 1:** Date of testing and outcome of the Chembio Dual Path Platform (DPP^®^) VetTB lateral flow assay performed on African elephant serum samples.

**Collection date**	**Chembio DPP**^**^®^**^ **VetTB (RLU)**		

	**Control line**	**Test line 1 (MPB83)**	**Test line 2 (CFP10/ESAT60)**
26 March 2015	126.5	0–Neg	0–Neg
13 April 2016	119.8	2.8–Neg	0–Neg
27 March 2018	94.7	0–Neg	22.4–Pos

Serum samples were tested according to the manufacturer's guidelines, and results quantified by a DPP optical reader in relative light units (RLU). Tuberculosis-specific antibody response was observed in 2018 prior to euthanasia, with the presence of circulating CFP10/ESAT-6-reactive antibodies. Test line results with RLU reading ≥ 5 were considered positive.

**Figure 1:**
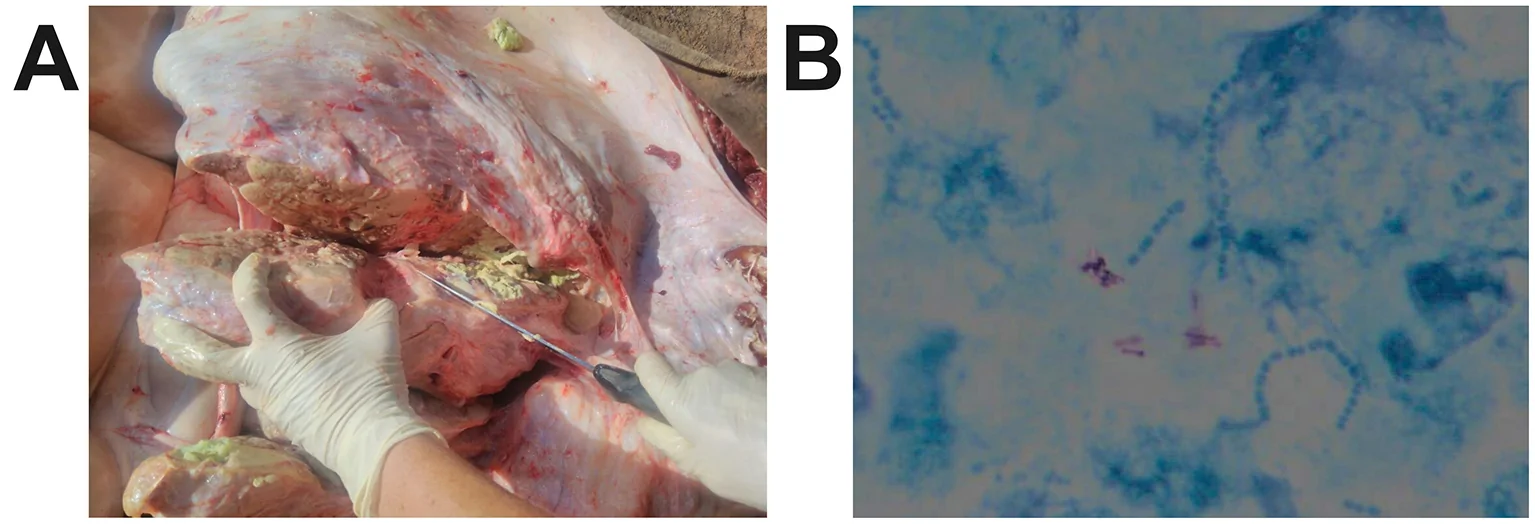
**(A)** Clinical signs of chronic pneumonia and extensive macroscopic lesions compatible with TB were observed in the elephant, confirming the presence of the disease. Multifocal to coalescing yellow-gray nodules, surrounded by connective tissue and central caseous necrosis were present in all pulmonary lobes. **(B)** Extra- and intra-cellular acid-fast rods within alveolar macrophages were visible after Ziehl-Neelsen staining.

At post-mortem, signs of chronic pneumonia and extensive macroscopic lesions compatible with TB were observed in the lung, confirming the presence of the disease (Figure 1). Multifocal to coalescing yellow-gray nodules, surrounded by connective tissue and central caseous necrosis were present in all pulmonary lobes. Extra- and intra-cellular acid-fast rods within alveolar macrophages were visible after ZN staining and quantified between scanty and 1+ according to the International Union Against Tuberculosis and Lung Disease (IUATLD) and World Health Organization (WHO) grading scales (33).

The GeneXpert MTB/RIF Ultra assay (Cepheid) detected the presence of *M. tuberculosis* complex DNA in the BAL, ETT, and lung tissue samples, while the TW sample resulted in an “MTB trace detected” outcome. No rifampicin resistance was reported by the assay. Mycobacterial growth was detected in all four samples (TW, BAL, ETT, and lung tissue) using conventional and Tika liquid culture methods, as well as ZN staining. The presence of *M. tuberculosis* was confirmed by RD-PCR from TW and ETT samples.

### Whole genome sequence analysis

For the two sequenced whole genomes obtained from the ETT cultures, more than 2 million reads were generated, with a mapping percentage to the *M tuberculosis* H37Rv reference genome exceeding 99% and a mean coverage of 100x and 129x, respectively (Table 2).

**Table 2:** Whole-genome sequencing data overview of *Mycobacterium tuberculosis* isolated from African elephant respiratory samples.

**Sample ID**	**Total reads**	**Mapped reads**	**Mapped reads (%)**	**GC-content**	**Mean coverage**
Endotracheal tube Tika	2,619,924	2,595,121	99.05	65.58	129
Endotracheal tube conventional MGIT	2,011,753	1,995,224	99.18	65.59	100

Statistics were generated with MTBseq and the *Mycobacterium tuberculosis* H37Rv sequence as reference using DNA extracted from mycobacterial culture of elephant respiratory samples (ETT).

The analysis of (sub-) lineage-associated markers revealed the presence of two distinct strains from different *M. tuberculosis* lineages in both samples. The predominant strain was classified as lineage 1.2.2.1 and a second strain was identified as lineage 4.9.1. No mutations associated with drug resistance were detected. A total of 30 lineage 1 and sub-lineage 1.2.2.1 specific genomic positions were interrogated for the presence of the expected and discordant alleles, respectively. Similarly, 20 positions specific for lineage 4 and sub-lineage 4.9.1 were included (Supplementary Table 1).

**Figure 2:**
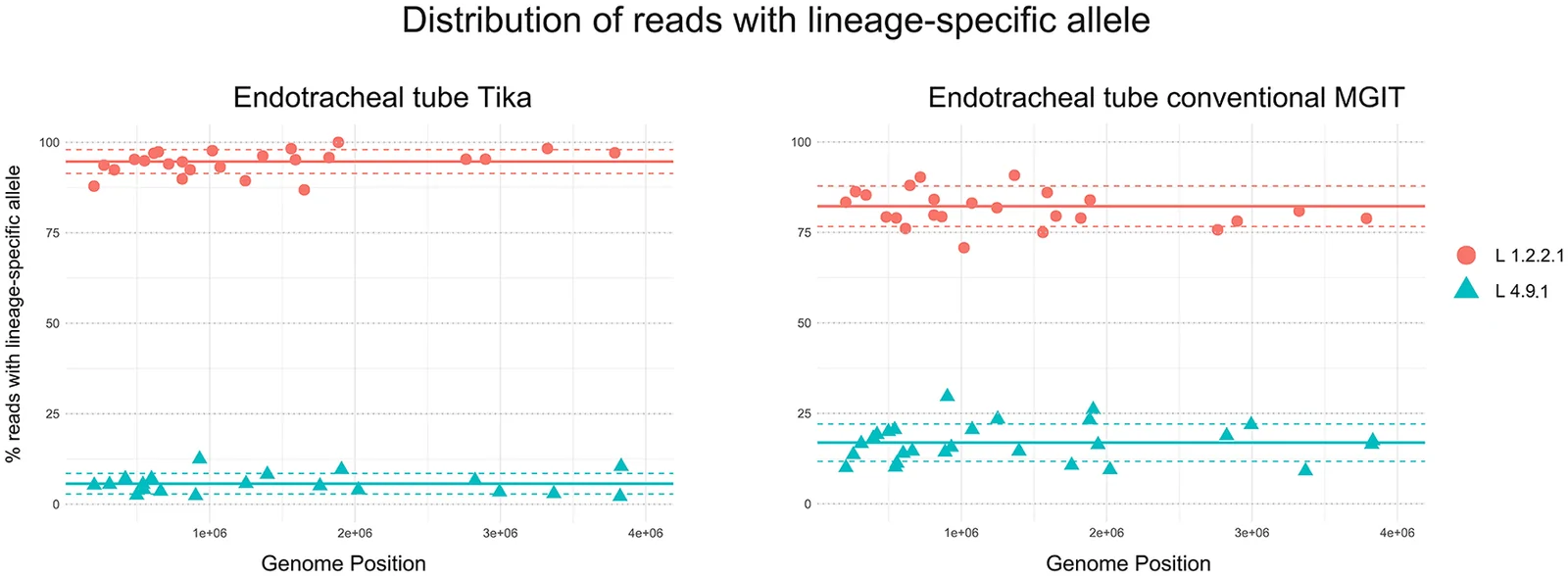
Whole genome sequencing of *Mycobacterium tuberculosis* isolates from an African elephant. Heterozygous SNP plots for one clinical sample obtained from African elephant endotracheal tube (ETT) fluid, cultured using two liquid culture media, Tika and conventional Mycobacteria Growth Indicator Tube (MGIT). The different allelic frequencies in lineage- and sub-lineage-defining genomic positions are illustrated. The x-axis represents contiguous SNPs across the genome (numbered sequentially) with heterozygous SNP calls, and the y-axis represents the percentage of non-reference alleles at that SNP level. A total of 30 lineage 1, sub-lineage 1.2.2.1 specific genomic positions were interrogated for the presence of the expected and discordant alleles, respectively. Similarly, positions specific for lineage 4 and sub-lineage 4.9.1 were included. Continuous lines represent the mean, and dotted lines represent the standard deviations of the percentages for each strain relative to the total number of reads.

The estimated fraction of the predominant strain (lineage 1.2.2.1) was determined to be 94.7% (SD = 3.3) and 82.2% (SD = 5.6) of the *M. tuberculosis*-mapped reads in the sequences obtained from Tika and conventional MGIT cultures, respectively. The underlying strain, identified as lineage 4.9.1, represented 5.7% (SD = 2.8) of the reads in Tika and 16.9% (SD = 5.2) in conventional MGIT cultures, respectively (Figure 2). To rule out contamination with the laboratory strain *M. tuberculosis* H37Rv, which also belongs to lineage 4.9, we manually inspected and compared 10 sub-lineage 4.9.1-defining genomic positions (Supplementary Table 1) between our sequences, the reference genome NC_000962.3, and a recently published laboratory reference strain (ERR3299635) from Stellenbosch University (34). All inspected positions in the elephant sequences displayed the lineage-specific alleles at the expected frequency, allowing confident differentiation between the clinical and laboratory strains.

The statistical tool designed to compare sequences by applying a GMM approach to distinguish different strain lineages' fractions confirmed the presence of distinct *M. tuberculosis* strains. The proportion of reads belonging to lineage 1.2.2.1 was estimated to be 92.4% (SD = 1.2%) and 78.6% (SD = 2.5%) of the *M. tuberculosis*-mapped reads in the sequences obtained from Tika and conventional MGIT cultures, respectively. The remaining reads were classified as lineage 4.9.1 (Supplementary Figures 1A, B).

## Discussion

Both African elephants and Asian elephants are classified as endangered species (35, 36). One of the leading causes of mortality in African elephants is poaching for ivory (37), whereas habitat fragmentation and human-elephant conflict are the main reasons for the current population decline in Asia (38, 39). In addition, TB has emerged as a potential threat to wildlife conservation, including African and Asian elephants (9, 40–42). The transmission dynamics and implications of *M. tuberculosis* infections in animal populations remain largely unknown. Individual strains of *M. tuberculosis*, for example, those grouped in specific lineages such as L2, have been more frequently associated with drug resistance and transmission (12). However, the extent to which these strains can infect and transmit among animal hosts, particularly in endangered wildlife species like elephants, remains largely unknown and warrants further investigation to mitigate the pathogen's impact.

The exact timing of TB infection is extremely challenging to determine retrospectively, particularly in animals where subtle changes in fitness due to disease are not reported, and because there is great variability in the time from infection to progression to disease. Serial serological testing using the Chembio DPP^®^ VetTB assay revealed seroconversion between 2016 and 2018. However, the infection may have occurred earlier, with antibodies remaining undetectable until the disease progressed to a more advanced stage (43). Additionally, in the present case, two infection events with distinct strains have occurred. MSIs denote an infection wherein multiple unrelated strains, which did not evolve from an initial infecting strain, coexist within a single host concurrently (17). It is crucial to distinguish between MSI and microevolution, where microevolution arises from a single infection event and subsequent polyclonal diversification of the pathogen over time. *M. tuberculosis* MSIs have been reported in humans with discordant drug susceptibility profiles on more than one occasion (17). In animals, however, MSIs with members of the *M. tuberculosis* complex are rare and to the authors' knowledge, there is only one report of *M. tuberculosis* MSI in two working Asian elephants from Nepal (44). Paudel et al. used spoligotyping and large sequence polymorphism to identify mixtures of two distinct *M. tuberculosis* strains. In one elephant, they identified a combination of Indo-Oceanic and East African–Indian (CAS-Delhi) lineages, while in the second elephant, they found a mix of Indo-Oceanic and East Asian (Beijing) lineages.

The present findings underscore the high susceptibility of elephants to *M. tuberculosis* infection. Notably, both *M. tuberculosis* lineage 1 and, particularly, lineage 4 have been identified in a significant proportion of human TB cases in South Africa and Zimbabwe, both of which are high TB-burden countries (45–47). Our findings highlight the crucial role of NGS methods in detecting and differentiating TB-causing agents in elephants. Traditional methods, such as serologic assays and visual assessments of gross and histopathological lesions, are insufficient for distinguishing between *Mycobacterium bovis* and *M. tuberculosis* infections. Genome sequencing offers the significant strength of providing high-resolution data that enable the detection of mixed infections, an aspect that traditional genotyping methods, such as spoligotyping or speciation alone, cannot achieve (48). However, these techniques come with higher costs, dependency on culture, and the need for substantial data management and bioinformatics expertise to interpret the results effectively. While the investment in these advanced methods can be considerable, their ability to deliver comprehensive insights into complex infections makes them invaluable in modern microbial research (48).

The origins of these mixed infections are currently unidentified and could potentially derive from either humans or other animals kept in close proximity and harboring these specific lineages. Individuals with regular, close interactions with infected elephants, such as elephant keepers or mahouts, represent a plausible human reservoir for transmission. In conservation areas and zoological gardens, where elephants may have indirect contact with humans through visitors feeding the animals or direct contact with staff and zookeepers, especially in high TB burden regions like African and Asian range countries, it is crucial to employ comprehensive surveillance and diagnostic tools to accurately identify these pathogens. Collecting multiple samples from different body sites and lesion localizations is crucial for veterinarians and wildlife managers tasked with monitoring the health of elephants (49). This comprehensive approach enhances the reliability and relevance of epidemiological studies, ultimately contributing to more effective TB management and control strategies in both elephant and human populations. Additionally, the monitoring of staff members working closely with susceptible animals in conservation parks and zoological gardens needs to be re-evaluated and standardized. Currently, screening for potential human TB reactors typically occurs reactively, after an outbreak, for contact tracing purposes, and to initiate prophylactic treatment (50). However, adopting an enhanced biosecurity approach, including regular testing of personnel involved, could prevent infections in both animals and staff, thereby reducing the incidence of fatalities resulting from zoonotic and reverse zoonotic events. This single case study provides a unique insight into the mixed *M. tuberculosis* infection in a captive elephant; however, it does not allow for broader generalization regarding the infection's timing and route of transmission. Additionally, the rarity of such cases and the lack of investigation into the elephant's immune status, potential human contacts, other animals within the facility, and environmental factors highlight the need for further research in this area.

## Conclusion

This study aimed to report the serological, genomic, and epidemiological characteristics of a human-managed African elephant affected by tuberculosis. We address the diagnostic challenges, particularly focusing on the capability of WGS to detect the presence of mixed *M. tuberculosis* infections and to estimate the proportion of the different populations from liquid cultures is demonstrated.

## Data Availability

The datasets presented in this study can be found in online repositories. The names of the repository/repositories and accession number(s) can be found in the article/Supplementary material.
