# Supplementary material for: Implications of serological, genomic, and epidemiological insights into Mycobacterium tuberculosis mixed infection in a human-managed African elephant (Loxodonta africana)
Source: Front Tuberc. 2025 Jan 20;2:1484394. doi: 10.3389/ftubr.2024.1484394 (PMC13494983; doi:10.3389/ftubr.2024.1484394)
Supplement: Supplementary file 1 [file Supplementary_file_1.docx]

Supplementary Figures

A	B

**Supplementary Figure 1.** **Alternative allele distribution derived from mixed populations of *Mycobacterium tuberculosis* estimated using the Gaussian Mixture Model (GMM)-based statistical tool GMM4TB.** Respiratory samples from the endotracheal tube (ETT) of a zoo-kept African elephant in South Africa were cultured using **A**) an improved mycobacterial culture method (TiKa Diagnostics, London, UK) and **B**) conventional MGIT media. The proportion of reads belonging to lineage 1.2.2.1 was estimated to be 92.4% (SD = 1.2%) and 78.6% (SD = 2.5%) of the *M. tuberculosis*-mapped reads in the sequences obtained from Tika and conventional MGIT cultures, respectively. The remaining reads were classified as lineage 4.9.1.
